# Mixture of quantum dots and ZnS nanoparticles as emissive layer for improved quantum dots light emitting diodes†

**DOI:** 10.1039/c9ra01462d

**Published:** 2019-05-15

**Authors:** Taeyoung Song, Jun Young Cheong, Hyunjin Cho, Il-Doo Kim, Duk Young Jeon

**Affiliations:** Department of Materials Science and Engineering, Korea Advanced Institute of Science and Technology 291 Daehak-ro, Yuseong-gu Daejeon 305-701 Republic of Korea dyjeon@kaist.ac.kr

## Abstract

Recently, quantum dots based light-emitting diodes (QLEDs) have received huge attention due to the properties of quantum dots (QDs), such as high photoluminescence quantum yield (PLQY) and narrow emission. To improve the performance of QLEDs, reducing non-radiative energy transfer is critical. So far, most conventional methods required additional chemical treatment like giant shell and/or ligands exchange. However that triggers unsought shifted emission or reduced PLQY of QDs. In this work, we have firstly suggested a novel approach to improve the efficiency of QLEDs by introducing inorganic nanoparticles (NPs) spacer between QDs, without additional chemical treatment. As ZnS NPs formed a mixture layer with QDs, the energy transfer was reduced and the distance between the QDs increased, leading to improved PLQY of mixture layer. As a result, current efficiency (CE) of the QLED device was improved by twice compared with one using only QDs layer. This is an early report on utilizing ZnS NPs as an efficient spacer, which can be utilized to other compositions of QDs.

## Abstract 2

Mixture of quantum dots and ZnS nanoparticles as emissive layer for improved QLEDs by decreasing energy transfer between the QDs.

## Graphical abstract

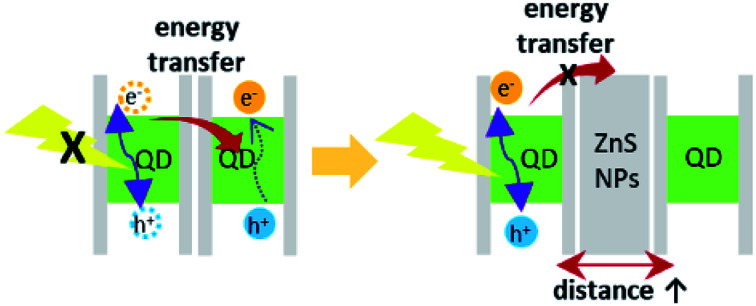

## Introduction

A number of two-dimensional layered materials have received considerable attention in many fields such as nanoelectronics and optoelectronics.^1–6^ Among the various materials, the quantum dots (QDs) have been vigorously investigated because of their unique characteristics, including bandgap, electrical and optical property change depending on their decreased size under exciton Bohr radius.^7^ Due to the bandgap controlled by size, QDs with band-edge emission can radiate a broad spectrum of light from the ultraviolet to infrared region.^8^ Additionally, QDs have not only high photoluminescence quantum yield (PLQY) due to the core/shell structure but also narrow emission.^9–12^ Based on these characteristics, QLEDs have been studied as a replacement for organic light-emitting diodes in next-generation display devices.^13–15^

Effort has been made to improve the performance of QLEDs by optimizing device architectures using new types of charge transport layers for the efficient injection of holes and electrons from respective electrodes to the QD emissive layer (EML).^15–17^ Although the EML has received less attention, this layer needs to be studied from the point of view of non-radiative recombination of excitons.^18–21^ The EML consists of close-packed QDs, which is in contrast to QDs dispersed in non-polar solution. The EML structure induces energy transfer among QDs, including Förster resonant energy transfer (FRET).^13,22–24^ FRET is a non-radiative recombination process that limits the efficiency of devices.^18–21^

Thus far, to reduce non-radiative energy transfer, the distance between QD cores has been engineered according to shell thickness control and/or exchange of ligands with various lengths.^18,21,23,24^ However, such methods are accompanied by additional chemical treatment of the original QDs. In the case of shell thickness control, the high temperature that is required for shell growth triggers diffusion of ions in QDs. This leads to a shifted photoluminescence (PL) peak and reduced PLQY of QDs. In addition, the ligand exchange processes decrease the PLQY of QDs.^25–27^ Therefore, any methods of longer distance between QDs are needed for higher efficiency of QLEDs, except for additional chemical treatments of QDs.

Herein, we firstly suggest that the efficiency of QLED devices is improved using an inorganic NP spacer between QDs without additional chemical treatment of the QDs. Inorganic nanoparticles (NPs) consist of ZnS that is generally used for the shell of type-I core/shell heterostructured QDs because it has a larger bandgap than the core of the QDs (∼3.6 eV).^28^ In order to apply ZnS NPs to the EML of QLEDs, we mixed ZnS NPs with green-emitting CdZnSeS/ZnS QDs dispersed in non-polar solvent. The solution was then applied to the EML of QLEDs by simple spin-casting.

The formed mixture layers of the QDs and ZnS NPs showed that the non-radiative energy transfer was reduced due to ZnS NPs acting as spacers increasing the distance between the QDs.^29–31^ Consequently, the PLQY of the mixture layers was improved with increasing ratio of ZnS NPs, and the current efficiency (CE) of the QLED device with the mixture layer was enhanced by about two times compared with only the QD layer. This simple structure would be applicable to not only CdZnSeS/ZnS QDs but also to QDs with various compositions due to the large bandgap of ZnS NPs.^31,32^

## Experimental

### Materials

Oleic acid (OA, 90%), 1-octadecene (1-ODE, 90%), trioctylphosphine (TOP, 97%), zinc acetate (99.99%), sulfur (99.998%), zinc diethyldithiocarbamate (Zn DDTC, 97%), ZnO (99.999%), cadmium acetate (99.995%), Se powder (99.99%) zinc acetate dihydrate (≥98%), lithium hydroxide monohydrate (≥98%), and poly[(9,9-dioctylfluorenyl-2,7-diyl)-*co*-(4,4′-(*N*-(4-*sec*-butylphenyl)diphenylamine))] (TFB) were purchased from Sigma Aldrich.

### Synthesis of green-emitting CdZnSeS/ZnS quantum dots

Although the band-edge emission of the QDs can be changed by using only the size effect, the quantum dots have possibility of low photoluminescence efficiency or low stability due to increased surface to volume ratio. Hence, we adopted the CdZnSeS/ZnS quantum dots of alloy structure containing additional Zn and S.^33,34^ The synthetic method of the QDs is as follows. 277 mg of ZnO, 32 mg of cadmium acetate, 15 mL of 1-ODE and 7 mL of OA were loaded in three-neck flask and heated to 130 °C for 1 h under high vacuum. Subsequently, after increasing temperature to 320 °C for colorless and clear solution under Ar atmosphere, the solution was cooled to 300 °C. Then 158 mg of selenium and 64 mg of sulfur powder dissolved in 2 mL of TOP was quickly injected into the flask. After 10 min, the flask was cooled to 225 °C and 200 mg of Zn DDTC dissolved in 2 mL of TOP was injected into the solution. Next, the solution was heated to 280 °C and maintained for 30 min.

### Synthesis of ZnS NPs

289 mg of sulfur, 219 mg of zinc acetate, 6 mL of OA, and 6 mL of 1-ODE were loaded into 100 mL three-neck flask. After being degassed under high vacuum for 1 h at 50 °C, the solution was heated to 300 °C and maintained for 60 min under Ar atmosphere.

### Synthesis of ZnO NPs

ZnO NPs were synthesized based on a previous paper.^35^ 439 mg of zinc acetate dihydrate was added in 20 mL of absolute ethanol and the solution was heated to 80 °C for 1 h with stirring. After homogeneous and transparent solution was obtained, 118 mg of lithium hydroxide monohydrate was added and the solution was sonicated for 1 h to obtain ZnO NPs.

### Device fabrication

Patterned ITO glass substrates were sonicated in three different solvents (acetone, methanol and isopropyl alcohol) for 30 min and dried at 120 °C in oven and then treated with UV–ozone for 10 min. Poly(3,4-ethylenedioxythiophene):poly-(styrenesulfonate) (PEDOT:PSS, Clevios P VP AI4083) was spun-cast on the ITO substrate with a spin-rate of 3000 rpm for 45 s, followed by baking at 180 °C for 1 h. TFB layers were casted on the PEDOT:PSS from solution (8 mg mL^−1^ in *p*-xylene) with spin-rate of 3000 rpm for 45 s and then annealed at 180 °C for 1 h. The mixture solutions of QDs and ZnS NPs (20 mg mL^−1^ in octane) were spun-cast directly on TFB layer with spin-rate of 3000 rpm for 45 s and dried at 100 °C for 30 min. On the mixture layers, the ZnO NPs (40 mg mL^−1^ in 2-methoxyethanol) were formed with spin rate of 2000 rpm for 45 s. Finally, Al cathode layer of 120 nm thickness was deposited by thermal evaporation at base pressure of 1 × 10^−7^ torr for complete devices.

### Characterization

Morphological features were obtained using atomic force microscope (AFM, Innova-LabRam HR 800, Bruker and Horiba), scanning electron microscope (SEM, SU8230, Hitachi) and field emission transmission electron microscopy (TE-TEM, Tecnai G2 F30 S-Twin). Energy dispersive X-ray spectroscopy (EDS) mapping images were observed using SEM with EDS. Fourier transform infrared (FTIR) spectroscopy, thermogravimetric (TG) analysis and X-ray fluorescence (XRF) analysis were conducted using Nicolet iS50 of Thermo Fisher Scientific Instrument, TG209F1 Libra of Netzsch, and ZSX Primus II of Rigaku, respectively. The photoluminescence spectra were recorded using FL920, Edinburgh instruments, and F-7000, Hitachi. X-ray diffraction (XRD) patterns were obtained by a X-ray diffractometer (SmartLab, Rigaku). The decay times of photoluminescence were recorded by FL920 with picosecond pulsed diode laser of 470 nm, EPL-470, and the values were calculated using a F900 program. The decay times of layers were measured at 528.2 nm at room temperature. For the QLED, electron only and hole only devices, the current–luminance *versus* driving voltage results were obtained by Konica Minolta spectroradiometer (CS-2000) and a Sourcemeter (Keithley 2635A). X-Ray photoelectron spectroscopy (XPS) data and ultraviolet photoemission spectroscopy (UPS) spectra were obtained using K-alpha (Thermo VG scientific) and Axis-Supra (Kratos), respectively.

## Results and discussion

**Fig. 1:**
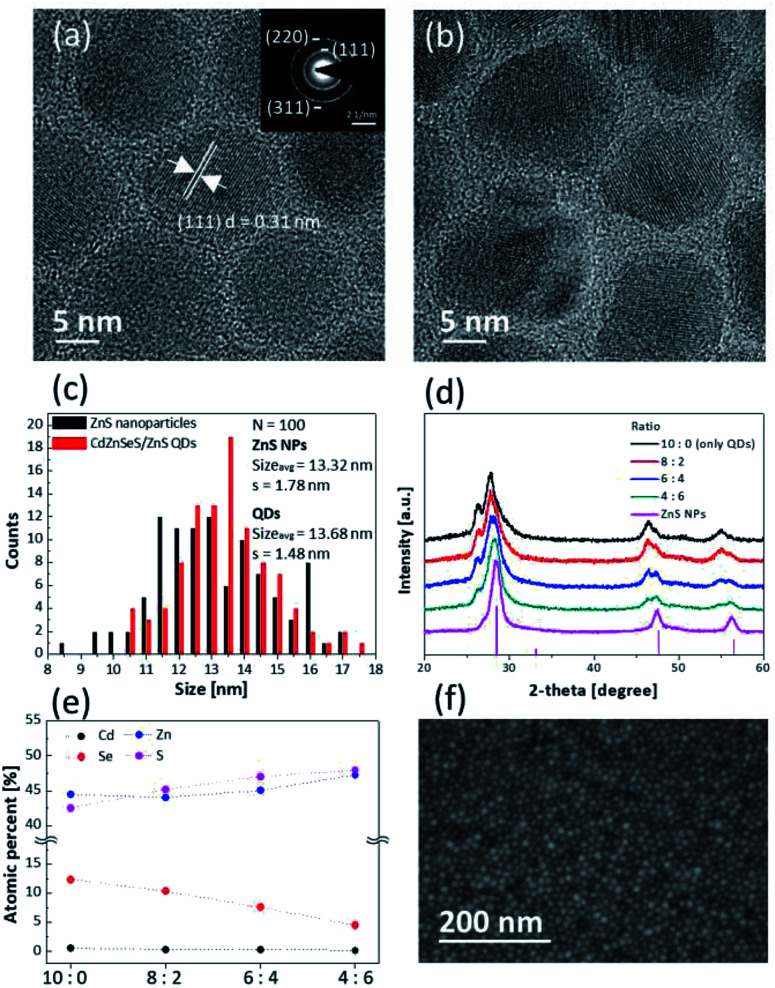
(a) High-resolution TEM image and SAED pattern (inset) of ZnS NPs. (b) High-resolution TEM image of green-emitting CdZnSeS/ZnS QDs. (c) Size distributions of the ZnS NPs and the QDs. *N* and *σ* in the figure are number of samples and sample standard deviation, respectively. (d) XRD patterns of the mixture layers coated on glass depending on ratios of the QDs to the ZnS NPs (peaks at the bottom: zinc blende structure of ZnS (ICDD no 01-077-3378)). (e) Atomic percentages of elements by XPS analysis. (f) SEM image of surface of the layer of 6 : 4.

Fig. 1a and b show high-resolution TEM images of the synthesized ZnS NPs and CdZnSeS/ZnS QDs, respectively. Low-resolution TEM images of ZnS NPs and QDs are shown in Fig. S1.† In Fig. 1d, the XRD pattern shows that the ZnS NPs are a zinc blende structure (ICDD no 01-077-3378), and the selected area electron diffraction (SAED) pattern also indicates that the ZnS NPs possess a zinc blende structure and high crystallinity (Fig. 1a, inset). Additionally, the SAED pattern shows that three out of five diffraction rings near the centre were indexed to the same positions as (111), (220), (311). Fig. 1a shows that the ZnS NPs have lattice fringe spacing parallel to ∽0.31 nm and this was assigned to the d-spacing of (111) plane of zinc blende ZnS.^36^Fig. 1d also shows the XRD pattern of the synthesized green-emitting CdZnSeS/ZnS QDs at 10 : 0 (only QDs). The detailed XRD pattern of the QDs, which have an alloy core structure and ZnS shell structures, is shown in Fig. S2.†

The average sizes of the ZnS NPs and QDs were 13.32 nm and 13.68 nm, respectively (Fig. 1c). The synthesized ZnS NPs were similar to QDs with about 3% difference to exclude variables for size and capping ligands. The role of a ligand is to remove surface defects and improve colloidal dispersion stability in solvent. The synthesized ZnS NPs were capped with the same oleic acid ligand that was used for the green-emitting CdZnSeS/ZnS QDs.^37^ In Fig. S3,† the C–H vibrations of the CH_2_ group and the COO^−^ vibrations of the carboxylic group, which belong to the oleic acid ligand, were confirmed using FTIR spectroscopy. It was identified that the carboxylate anion formed chemical bond with the Zn cation on the surface.^38,39^ Organic ligand materials have a lower evaporation temperature than inorganic ones, and TG analysis was conducted for checking the proportion of ZnS NP ligands. At temperature over 450 °C, all of the organic ligands were evaporated, and the content was about 16%, which is similar to that of the QD ligands (Fig. S4†). In addition, XRF analysis showed about 13% of the carbon content in the synthesized ZnS NPs and this result is similar to that obtained by TG analysis (Table S1†). The ZnS NPs and QDs would be dispersed in the same non-polar solvent.

Octane was chosen as the solvent to analyse the characteristics of the mixture of the QDs and ZnS NPs because it has already been used as an orthogonal solvent for quality improvement of EML in QLEDs.^40^ The mixture layer was deposited by spin-casting of the solution mixed with the QDs and ZnS NPs. Solution concentration was controlled to 20 mg mL^−1^ and ratio of the QDs to ZnS NPs was set to 10 : 0 (only QDs), 8 : 2, 6 : 4, and 4 : 6 according to weight percent of these. Fig. 1d shows the XRD patterns of the mixture layers coated on glass with different ratios. When the layer of 10 : 0 was formed by only QDs, Fig. 1d shows that the XRD peaks gradually shifted to peaks assigned to the ZnS structure, and it was confirmed that the mixture layers were well blended in terms of macroanalysis of the XRD. SEM-EDS mapping images also show uniform distribution of the QDs and ZnS NPs for the layer of 6 : 4 (Fig. S5†). XPS analysis showed that the proportion of cadmium and selenium decreased contrary to increasing proportion of zinc to sulfur depending on increasing the ratio of ZnS NPs (Fig. 1e). The detailed data of atomic percentages are shown in Table S2.†

In Fig. 1f, the SEM image shows the uniform surface of the mixture layer of 6 : 4. Although the layer consists of two different materials, it was identified that inhomogeneous aggregations of each particle were not observed in the other layers as well as the layer of 6 : 4 (Fig. S6†). In addition, the surface roughness of the layers became smoother after mixing of the QDs with the ZnS NPs because the QDs were mixed with smaller sized-ZnS NPs that fill empty space in the QD layer (Fig. S7†). This means that the uniform mixture layer is more adequate to apply it as the EML of QLEDs for which the layer quality is important.^41–43^

**Fig. 2:**
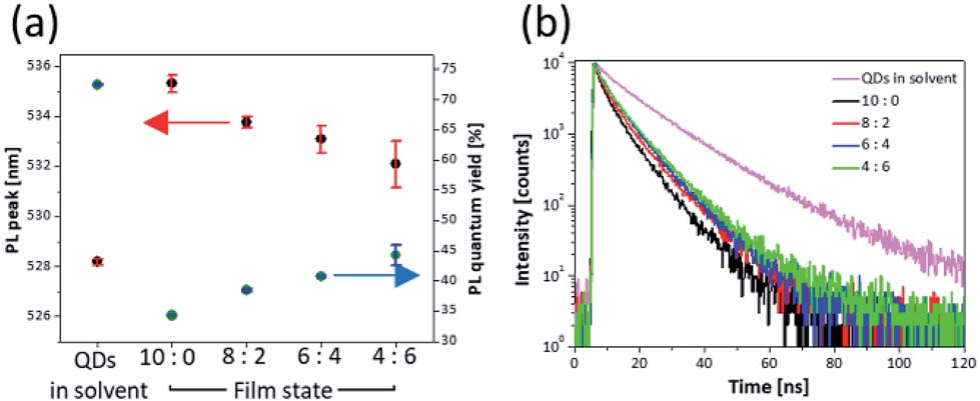
(a) PL peaks, PLQYs and (b) transient PL decay curves corresponding to four different ratios of the QDs to the ZnS NPs.

**Fig. 3:**
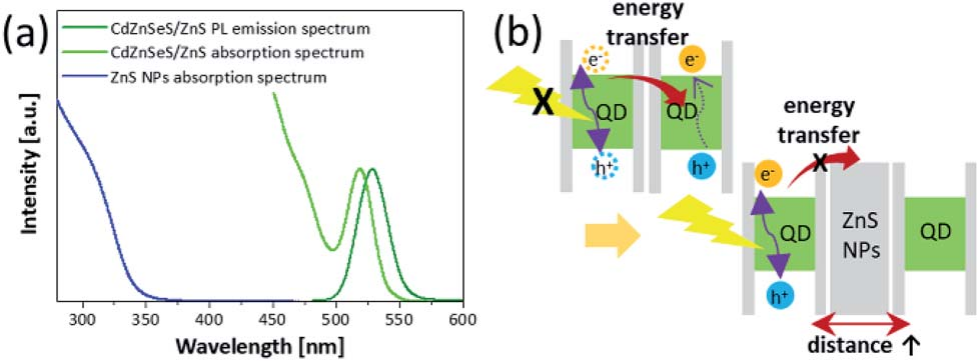
(a) PL and absorption spectra of the QDs and absorption spectrum of the ZnS NPs. (b) Schematic illustration on the ZnS NPs that block the energy transfer.

The PL properties of each mixture layer are shown in Fig. 2 and Table 1. The layer of 10 : 0 (only QDs) exhibited a red-shifted PL peak (535.3 nm) and reduced PLQY (34.3%) compared with the solution state of the QDs (528.2 nm, 72.5%) (Table 1). In the film state, a factor of the changed properties is non-radiative FRET between QDs. The FRET vigorously occurs by interdot coupling strength when the distance between the donor and the acceptor becomes closer, and spectra overlap of the donor emission and the acceptor absorption becomes greater.^22–24,30,31^ Because the synthesized QDs have considerably large aforementioned overlap of the spectra due to small Stoke's shift as shown in Fig. 3a. When the QDs are close to each other, the large spectral overlap gives rise to not radiative recombination of excitons formed in the QDs but excitation of other QDs in close proximity and non-radiative quenching of excitons.^8^ Consequently, the FRET is accompanied by the red-shifted PL peaks and reduced PLQYs in the QD layer where the distance between QDs is extremely short compared with that in the solution state. Although the FRET exists because of the intrinsic characteristics of the QDs, it should be eliminated for improved performance of QLED device. However, depending on increasing ratio of ZnS NPs, the PL peak and the PLQY gradually were recovered, and then the layer of 4 : 6 had a PL peak of 532.1 nm and PLQY of 44% (Fig. 2a). These properties show that the non-radiative recombination of energy transfer decreases due to presence of the ZnS NPs. Fig. 3b shows a schematic illustration of the role of the ZnS NPs. Because the large band gap of ZnS NPs starts to absorb wavelength below 350 nm, it is hard to absorb light emitted from the QDs. Hence the aforementioned spectral overlap decreases (Fig. 3a).^31,44^ In addition, as the ZnS NPs act as spacers increasing the distance between QDs, the ZnS NPs support the radiative recombination of excitons formed in QDs. In other words, the factors of suppressing FRET become favourable for the improved QLED performance due to using the mixture layer between the ZnS nanoparticles and the quantum dots. This also means that because the absorption region of ZnS NPs is in the UV range, the simple method would be sufficiently extended not only to the green-emitting CdZnSeS/ZnS QDs but also to other QDs emitting visible light.^32^

**Table 1:** Details on the PLQYs, PL peaks and decay times demonstrated in Fig. 2

	PLQY [%]	PL peak [nm]	Decay time [ns]
QDs in solution	72.5	528.2	13.9
10 : 0	34.3	535.3	6.1
8 : 2	38.5	533.8	6.8
6 : 4	40.8	533.1	7.1
4 : 6	44.3	532.1	7.8

Fig. 2b shows the decay time of films corresponding to the ratio of the QDs to ZnS NPs. The QD solution has 13.9 ns of decay time. However, the decay time of the layer of 10 : 0 drastically decreased to 6.1 ns by the aforementioned energy transfer, which is a faster reaction than the radiative recombination rate of excitons in QDs.^22,23,45^ However, as the ZnS NP content of the mixture layer increased, reduced decay time was recovered toward the solution state, and the 4 : 6 layer had 7.8 ns of decay time. Additionally, the energy transfer efficiency is calculated from using eqn (1).^46^ The energy transfer efficiency (*η*_FRET_) is1
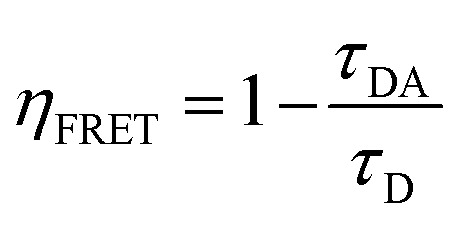
calculated by two factors, which are decay time (*τ*_DA_) of donors where are together with acceptors and a decay time (*τ*_D_) of donors without acceptors (Table S3†). In this experiment, the *τ*_DA_ was calculated by a decay time of film states and the *τ*_D_ was calculated for QDs in solution. It was assumed that the energy transfer between QDs did not occur in solution due to much longer distance. Consequently, the energy transfer decreased from 56.1% to 43.9% at the layer of 4 : 6 compared with the layer of 10 : 0, meaning the ZnS NPs were effective in eliminating energy transfer.

**Fig. 4:**
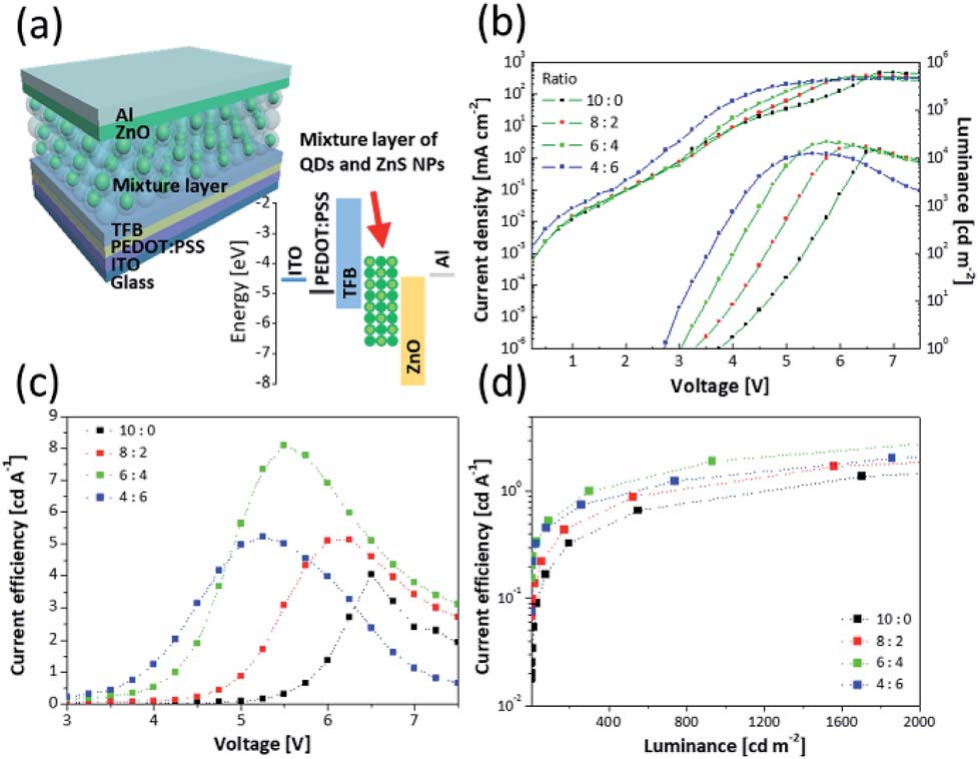
(a) Schematic device structure and energy level diagram. (b) *J*–*V*–*L* characteristics of QLEDs with different ratios of the QDs to the ZnS NPs. CE as a function of (c) voltage and (d) luminance for the QLEDs.

In order to apply the mixture layer as an EML of QLED, device performance was investigated using solution-processed QLEDs structure. This device adopted a normal structure and was fabricated by sequential deposition of PEDOT:PSS, TFB, the mixture layers, ZnO NPs, and finally an Al cathode on an ITO (anode) substrate. Table 2 summarizes the device performance of the QLEDs. Fig. 4a shows the architecture of the devices and corresponding energy levels. Fig. 4b and c show the current density–voltage–luminance (*J*–*V*–*L*) characteristics and CE depending on voltage for the QLEDs, respectively. Fig. 4d exhibits graphs of CE *vs.* luminance for QLEDs depending on the ratio of QDs to ZnS NPs.

**Table 2:** Device performances of the QLEDs using the mixture layer as an EML

	Current efficiency [cd A^−1^]		Max. luminance [cd m^−2^]	*V*_turn-on_ a [V]
	@1000 cd cm^−2^	Maximum		
10 : 0	0.91	4.04 @ 6.5 V	14 814	3.7
8 : 2	1.21	5.14 @ 6.25 V	18 064	3.3
6 : 4	2.06	8.10 @ 5.5 V	21 432	3.0
4 : 6	1.45	5.23 @ 5.25 V	12 401	2.7

a *V*_trun-on_ is defined at the luminance of 1 cd m^−2^.

Based on the results, devices incorporating the ZnS NPs exhibit significantly improved maximum CE. Specifically, the QLED of 6 : 4 showed a peak CE of 8.10 cd A^−1^, which is about twice compared with the QLED of 10 : 0. In addition, at the same luminance condition, all of the performances for QLEDs consisting of a mixture layer were higher than QLED consisting of only a QD layer. At 1000 cd cm^−2^, the QLED of 8 : 2 and 4 : 6 had CEs of 1.21 cd A^−1^ and 1.45 cd A^−1^, respectively. Notably, the QLED of 6 : 4 had CE of 2.06 cd A^−1^, which is about twice that of the QLED of 10 : 0 (0.91 cd A^−1^). The improved results are attributed to the ZnS NPs. Although the maximum luminance of QLEDs including ZnS NPs was improved, the QLED of 4 : 6 showed decreased maximum luminance (12 401 cd m^−2^) compared with the QLED of 6 : 4 (Fig. 4d). This is because the absolute quantity of QDs decreased as the content of ZnS NPs increased in the mixture layers.

**Fig. 5:**
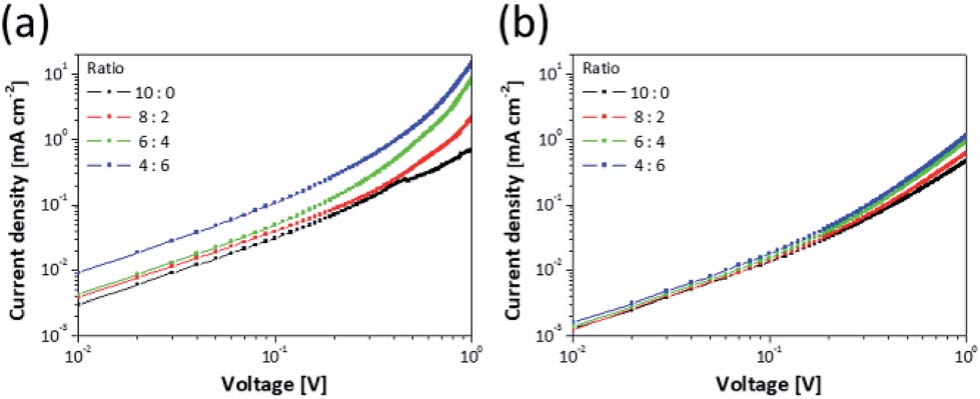
Current density–voltage graphs for (a) the EOD (Al/mixture layer/ZnO/Al) and (b) the HOD (ITO/PEDOT:PSS/TFB/mixture layer/MoO_3_/Al) corresponding to ratio of the QDs to the ZnS NPs.

Fig. 4b shows that the current density increased and the turn-on voltage (*V*_turn-on_) decreased corresponding to the content of ZnS NPs. Table 2 shows that although the *V*_turn-on_ was 3.7 V for the QLED of 10 : 0, it gradually shifted to 2.7 V for the QLED of 4 : 6. In addition, the voltages assigned to the CE peaks became lower (Fig. 4c). The CE peak that was 4.04 cd A^−1^ at 6.5 V for the QLED of 10 : 0 shifted to 5.23 cd A^−1^ at 5.25 V for the QLED of 4 : 6. This means that the ZnS NPs in the EML act not only spacers but also charge injection materials that transfer charges to QDs well. In order to identify the charges injection properties of ZnS NPs, an electron only device (EOD) and a hole only device (HOD) were fabricated. The EOD was fabricated by sequential deposition of Al, mixture layers, ZnO, and Al to move the electrons except for holes, and the HOD was fabricated by deposition of PEDOT:PSS, TFB, mixture layers, MoO_3_, and Al electrode for moving only holes. The energy level structures of EOD and HOD are shown in Fig. S8.† In Fig. 5, at low voltage range, due to insufficient energy for crossing the energy barrier of layers, the current density doesn't consist of injected charges from electrode but most charges existing inside layers of device. This region is governed by Ohm's law and called ohmic region.^15,47^ In the ohmic region of log *J*–log *V* curves, the current density is proportional to the voltage and has linear relationship (*J* ∝ *V*). As the voltage increases, a region where slope of the current density increases appears, and it is called space charge-limited current region. The current density of the region behaves with Mott–Gurney law and is dramatically increased by added charges from electrode.^48,49^

The EOD has a relatively larger current density than the HOD due to the difference of energy barrier between each charge transport layer to the QDs.^15^ Hence, Fig. 5a and b show that the EOD of 10 : 0 has a larger current density (0.031 mA cm^−2^ at 0.1 V) compared with the HOD of 10 : 0 (0.013 mA cm^−2^). Fig. S9† shows that although high occupied molecular orbital levels of mixture layer shifted to a deep level depending on increasing the ZnS NP portion, the current density gradually increased. In terms of charge mobility, the layers containing ZnS NPs have a higher mobility than only the QD layer due to the difference in energy levels of the core/shell heterostructure of QDs. This is because the ZnS shell of QDs has a larger bandgap than the core and acts as an energy barrier disturbing the movement of charges.^12,50,51^ Additionally, the current density of EOD was relatively more improved than that of HOD depending on the amount of contained ZnS NPs because the zinc blende structure of ZnS has an electron mobility of 180 cm^2^ V^−1^ s^−1^, which is more 36 times than a hole mobility (5 cm^2^ V^−1^ s^−1^) at 300 K.^52,53^ Based on the results, as the amount of ZnS NPs increased, the difference between the current densities of electrons and holes became bigger, and this induced the imbalanced charge injection to the QDs layer. Although the improved properties of charge injection contributed to low *V*_turn-on_, the imbalance increased. Hence, the QLED of 4 : 6 having a low absolute amount of QDs had lower CE compared with the other QLEDs incorporating ZnS NPs. This increment of current density affected the leakage current and current density of *J*–*V*–*L* curves (Fig. 4b).

## Conclusions

We successfully utilized inorganic ZnS NP as a spacer between QDs, which is different from the conventional approach of controlling the shell thickness and/or ligands of varying lengths. The introduction of ZnS NPs increased the PLQY of the mixture layers because ZnS NPs decreased the non-radiative recombination generated by energy transfer. In terms of the EML of QLEDs, the QLED consisting of the layer of 6 : 4 showed 8.10 cd A^−1^ of CE, which was two times higher than that of the QLED consisting of only a QD layer. The results suggest that inorganic ZnS NPs can be used as effective spacers to enhance the performance of QLEDs, which can also be extended to other QDs with different compositions due to a large band gap.

## Conflicts of interest

There are no conflicts to declare.

## Supplementary Material

RA-009-C9RA01462D-s001
