# Supplementary material for: Mixture of quantum dots and ZnS nanoparticles as emissive layer for improved quantum dots light emitting diodes
Source: RSC Adv. 2019 May 15;9(27):15177–83. doi: 10.1039/c9ra01462d (PMC9064264; doi:10.1039/c9ra01462d)
Supplement: RA-009-C9RA01462D-s001 [file RA-009-C9RA01462D-s001.pdf]

Electronic Supplementary Material (ESI) for RSC Advances.
This journal is © The Royal Society of Chemistry 2019

Supplementary Information

Mixture of Quantum Dots and ZnS Nanoparticles as Emissive Layer for Improved Quantum Dots Light Emitting Diodes

Taeyoung Song, Jun Young Cheong, Hyunjin Cho, Il-Doo Kim and Duk Young Jeon*

Department of Materials Science and Engineering, Korea Advanced Institute of Science and Technology, 291 Daehak-ro, Yuseong-gu, Daejeon 305-701, Republic of Korea.

E-mail : dyjeon@kaist.ac.kr

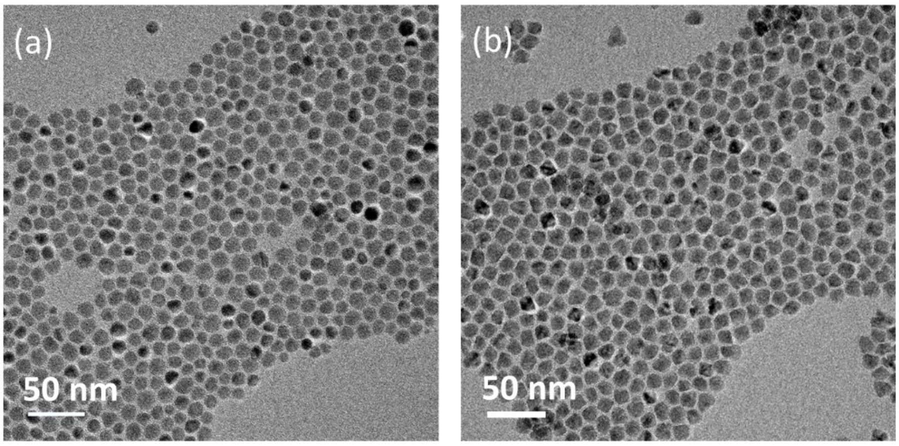

Fig. S1 TEM images of (a) the ZnS NPs and (b) the CdZnSeS/ZnS QDs.

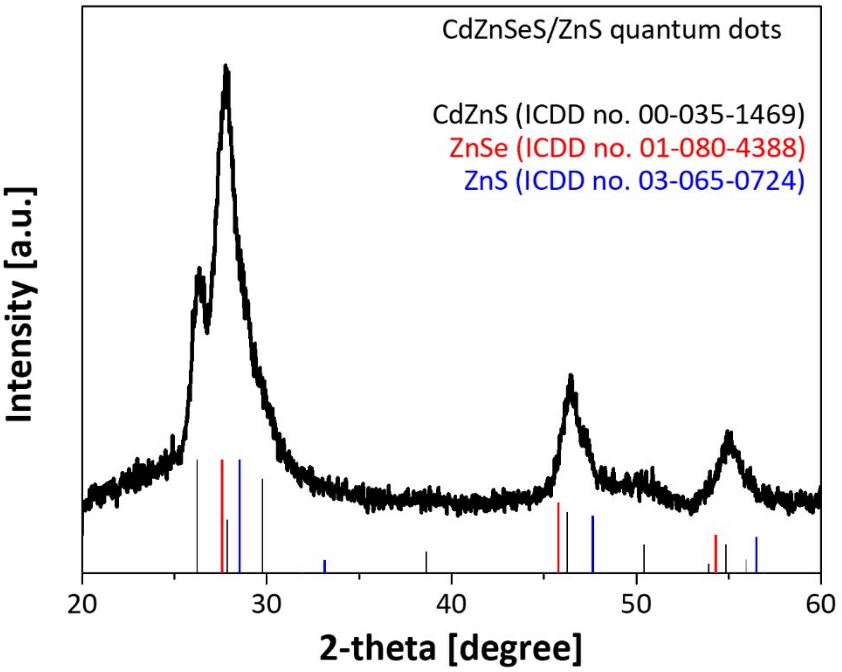

Fig. S2 XRD pattern of the CdZnSeS/ZnS QDs.

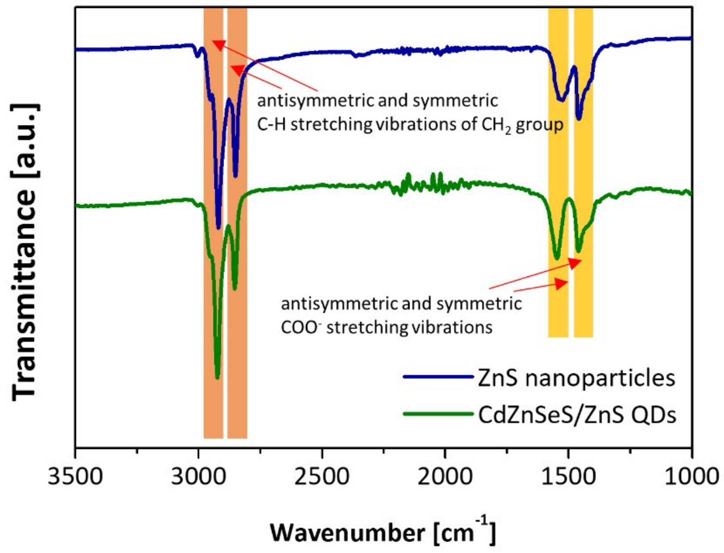

Fig. S3 FTIR spectra of the ZnS NPs and the QDs

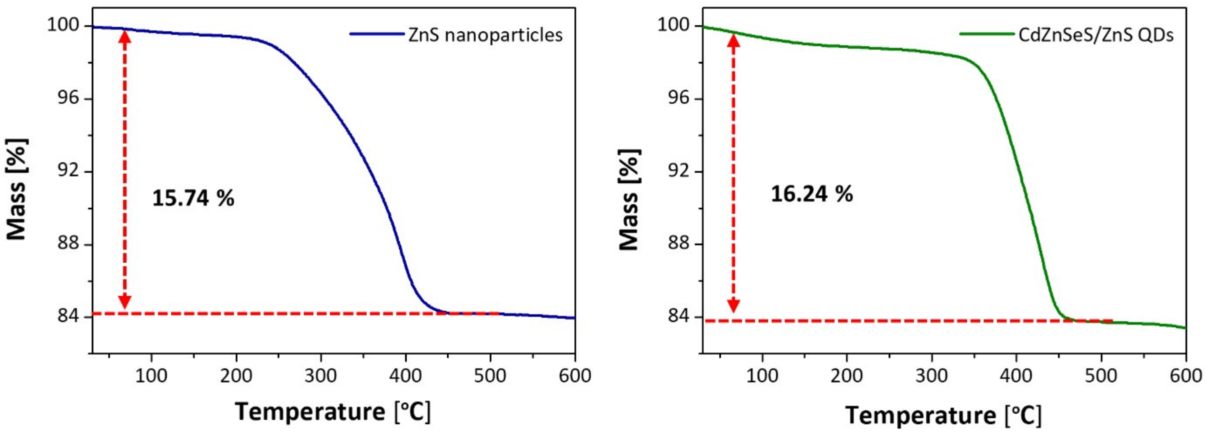

Fig. S4 TG analysis of the ZnS NPs and the QDs

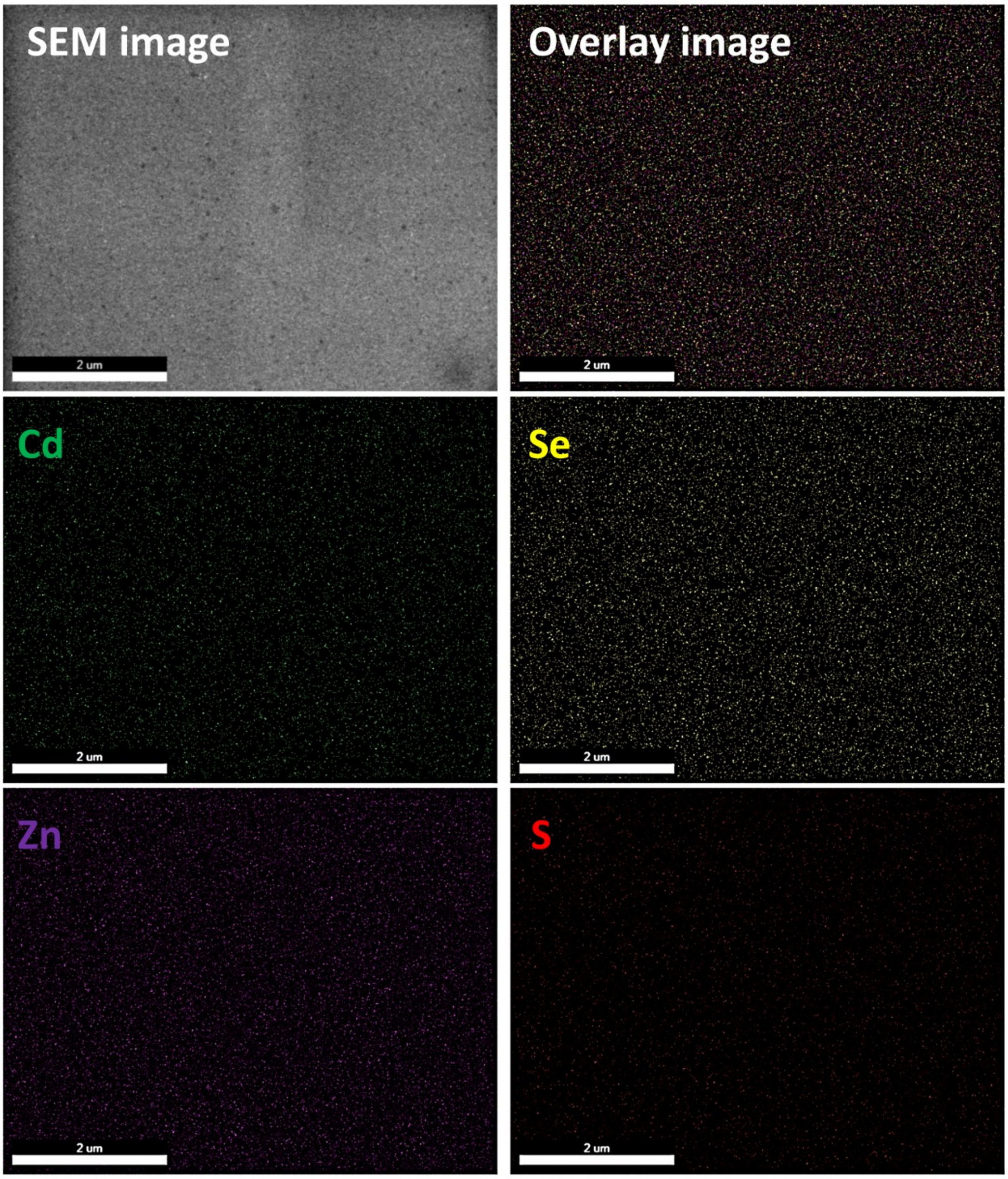

Fig. S5 EDS mapping of the layer of 6 : 4.

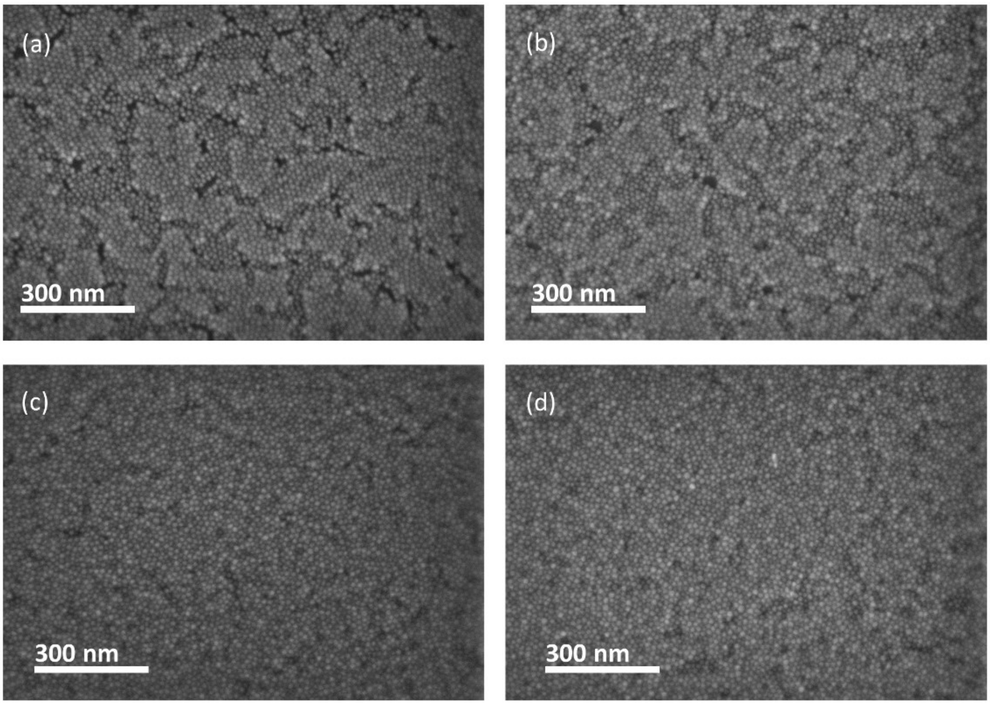

Fig. S6 SEM images of mixture film coated on silicon wafer by spin casting process : (a) 10 : 0, (b) 8 : 2, (c) 6 : 4, (d) 4 : 6.

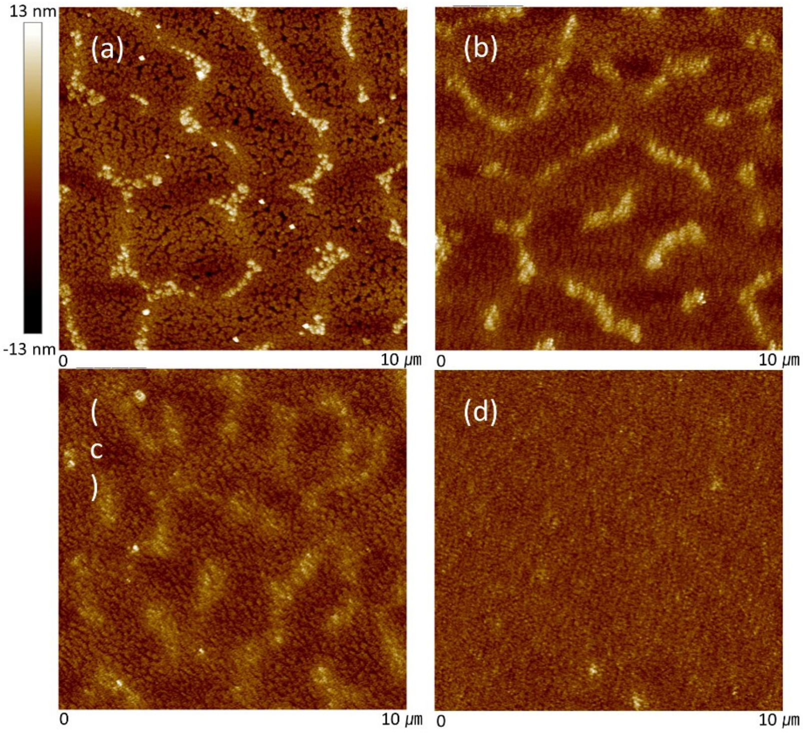

Fig. S7 AFM images of layer of (a) 10 : 0, (b) 8 : 2, (c) 6 : 4, and (d) 4 : 6 coated on silicon wafer. The root mean square (RMS) values are 2.80 nm, 2.09 nm, 1.84 nm, and 1.29 nm, respectively.

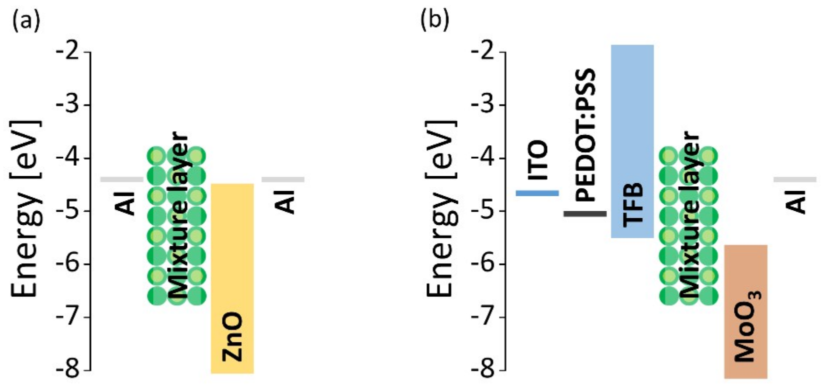

Fig. S8 The energy level structures of (a) EOD and (b) HOD

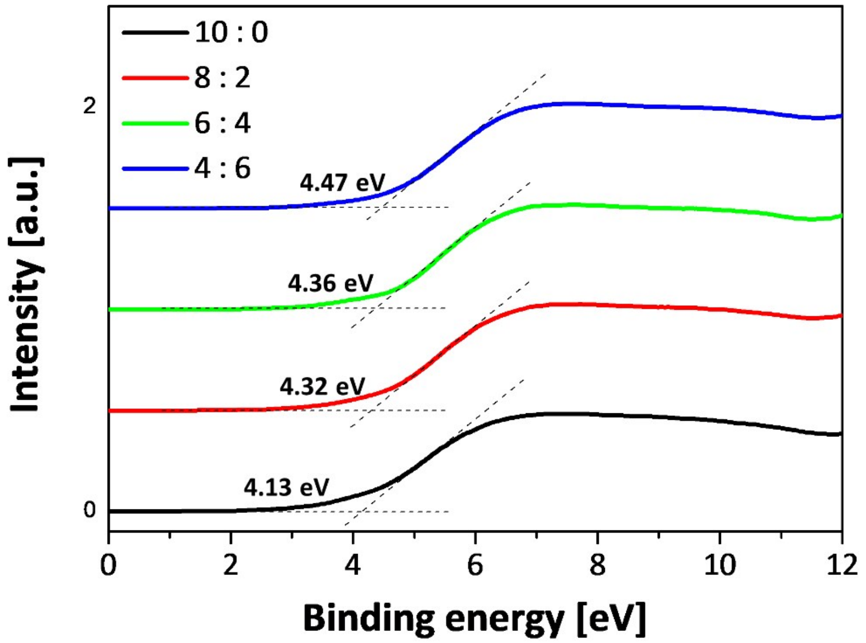

Fig. S9 UPS spectra of mixture layers on silicon wafer in HOMO region.

Table S1 Atomic percentages of elements on the ZnS NPs and the QDs using XRF analysis

	ZnS [wt%]	CdZnSeS/ZnS [wt%]
C	12.68	11.91
Zn	57.91	44.14
S	29.41	9.57
Cd	-	2.69
Se	-	31.69

Table S2 Atomic percentages of elements on the mixture films using XPS analysis.

	Cd [%]	Se [%]	Zn [%]	S [%]
10 : 0	0.58	12.42	44.47	42.53
8 : 2	0.37	10.37	44.07	45.19
6 : 4	0.29	7.61	45.03	47.07
4 : 6	0.24	4.56	47.28	47.93

Table S3 Decay time and FRET efficiency of the mixture films.

	decay time [ns]	FRET efficiency [%]
QDs in solvent	13.9	0
10 : 0	6.1	56.1
8 : 2	6.8	51.1
6 : 4	7.1	48.9
4 : 6	7.8	43.9
